# Elevated urinary urea by high-protein diet could be one of the inducements of bladder disorders

**DOI:** 10.1186/s12967-016-0809-9

**Published:** 2016-02-16

**Authors:** Ming Liu, Min Li, Jiangfeng Liu, Hongkai Wang, Dandan Zhong, Hong Zhou, Baoxue Yang

**Affiliations:** State Key Laboratory of Natural and Biomimetic Drugs, Department of Pharmacology, School of Basic Medical Sciences, Peking University, 38 Xueyuan Lu, Haidian District, Beijing, 100191 China; Department of Anatomy, Basic Medical College, Chongqing Medical University, Chongqing, 400016 China

**Keywords:** Urea, Protein-protein interaction network, Cytoscape, Inflammation, Cell cycle, Pathways in cancer, Bladder cancer

## Abstract

**Background:**

Previous work found that urea accumulation in urothelial cells caused by urea transporter B knockout led to DNA damage and apoptosis that contributed to the carcinogenesis. The purpose of this study is to explore the potential connection between high urinary urea concentration and the bladder disorders.

**Methods:**

A high protein diet rat model was conducted by feeding with 40 % protein diet. In-silico modeling and algorithm, based on the results of microarray and proteomics from the bladder urothelium, were used for the reconstruction of accurate cellular networks and the identification of novel master regulators in the high-protein diet rat model. Pathway and biological process enrichment analysis were used to characterize predicted targets of candidate mRNAs/proteins. The expression pattern of the most significant master regulators was evaluated by qPCR and immunohistochemistry.

**Results:**

Based on the analysis of different expressed mRNAs/proteins, 15 significant ones (CRP, MCPT2, MCPT9, EPXH2, SERPING1, SRGN, CDKN1C, CDK6, CCNB1, PCNA, BAX, MAGEB16, SERPINE1, HSPA2, FOS) were highly identified and verified by qPCR and immunohistochemistry. They were involved in immune and inflammatory response, cell cycle arrest, apoptosis and pathways in cancer. These abnormally activated processes caused the bladder interstitial congestion and inflammatory infiltrates under the thinner urothelium, cell desquamation, cytoplasm vacuolization, nucleus swelling and malformation in the high-protein diet group.

**Conclusions:**

We provided evidences that high urinary urea concentration caused by high-protein diet might be a potential carcinogenic factor in bladder.

## Background

As a malignant tumor of the urinary tract, urothelial bladder cancer (UBC) is the seventh most common malignancy in men and the seventeenth most common in women worldwide [1]. Globally each year, more than 400,000 new patients are diagnosed with bladder cancer and more than 150,000 patients die from it [2]. The survival rate of bladder cancer is poor, with almost 45 % of patients surviving for 5 years regardless of the type of treatment [3]. This heterogeneous disease has complex pathological mechanisms that are not yet fully expounded. So far, although it has been suggested that the risk of the disease is associated with many inducements including cigarette smoking, alcohol, drugs, chemical carcinogens and inflammation of the urinary tract, etc. [4], the exact causes of UBC have not been clarified [5].

Recent years, epidemiological studies showed that the incidence of the bladder cancer was the highest in the developed countries of Western Europe, North America and Australia but the lowest in Asian countries [6], which may be connected with their different diets and living habits. Meanwhile, results from genome-wide association studies (GWAS) had identified the urea transporter B (UT-B) gene (solute carrier family 14, member 1, *slc14a1*) as a new bladder cancer susceptibility locus in human [7, 8]. UT-B plays an important role in urine concentrating mechanism and it highly expresses in the urinary bladder urothelium [9, 10]. As far as we know, whether *slc14a1* variants had a direct effect on the urothelium or, possibly, the association with UBC was indirect through urine concentration or frequency of urination was not explicit yet [7]. An our previous study found that UT-B knockout caused urea accumulation in the bladder urothelial cells, which created an imbalance between the arginine-ornithine-polyamine pathway and the arginine-citrulline-nitric oxide pathway. These processes thus rendered urothelial cells more vulnerable to DNA damage and apoptosis that contributed to the bladder carcinogenesis [11]. These findings suggest that high urea concentration may serve as a potential pathogenic factor. Urea is a major nitrogenous waste product of protein metabolism. High-protein diet increases the urea concentration in urine, which makes us explore whether the continuous high urea stimulation could damage the urothelium and then increase the risk of carcinogenesis.

Here, using a high-protein diet rat model and a network-based strategy, it is found that high urinary urea concentration activated pathways including inflammatory response, cell cycle arrest, apoptosis and cancer in urothelium of rats with high-protein diet. These findings suggest a potential connection between high urinary urea concentration and the bladder disorder even carcinogenesis.

## Methods

### Ethics statement

All procedures in this study were carried out in strict accordance with the recommendations in the guide for the care and use of Laboratory Animals of China Association for Laboratory Animal Science. All animal care and protocols were approved by the Animal Care Committee of Peking University Health Science Center. All sacrifice was performed under pentobarbitone anesthesia, and every effort was made to minimize animal suffering.

### Animal model with high urea concentration and osmolality in the bladder

High-protein diet rat model in strictly controlled urine concentrating ability: Adult male SD rats (3 rats/group, body weight 160–180 g) were adapted in metabolic cages (Harvard Apparatus, Holliston, MA) for 3 days and fed with a standard synthetic rodent diet (20 % protein in diet). From day 4 to 7, the rats were fed with a diet with 40 % protein or 10 % protein in the high-protein diet group (HPD) or the low-protein diet group (LPD), respectively. In order to amplify the effects of urea concentration on the urothelial cells between the two groups, 3.5 μg/kg/12 h of dDAVP (1-deamino-8-D-arginine vasopressin), which could increase the absorption of water in the collecting duct and lead to a high urea concentration in the bladder, was administrated by subcutaneous injection in the HPD group and 10 ml/12 h water was intraperitoneally injected in LPD group from day 8 to 12.

High-protein diet rat model in normal urine concentrating ability: Adult male SD rats (6 rats/group, body weight 160–180 g) were separated into two groups: rats were fed with 40 % protein diet or 20 % protein diet in the HPD group or the normal-protein diet group (NPD) respectively for 40 days. In this model it made the urothelial cells of the HPD group suffer in a high urea concentration environment for a long time. During the whole study, the rats in the two models had free access to water and food.

The urinary osmolality was measured by freezing point depression (Micro-osmometer, FISKER ASSOCIATES, Norwood, MA). Urea concentration was measured with QuantiChrom Urea Assay kit (BioAssay Systems, Hayward, CA).

### Microarray analysis

Rat was deeply anesthetized with pentobarbital (85 mg/kg, i.p.) and the bladder was removed and cut into two parts. One was used for microarray analysis and the other was used for proteomics analysis. The urothelial cells were harvested by gently scraping the urothelium of bladder using a knife. Total RNA of the two groups was isolated with the RNA easy Isolation Kit (Qiagen). All RNA samples used in this experiment were of high quality as indicated by a ratio of 2:1 for 28S/18S rRNA and a ratio of >1.9 for OD_260_/OD_280_. The gene expression profiles were determined using GeneChip^®^ rat Gene 2.0 ST Arrays (Affymetrix). Samples were hybridized onto array chips according to Affymetrix protocol. The chips were washed and stained in a GeneChip Fluidics Station 450 and fluorescence detected with a GeneChip Scanner 3000 7G using the Affymetrix GeneChip Operating Software. Differentially expressed genes were identified as fold change ≥1.5 and P value <0.01 by unpaired *t*-test.

### Proteomics analysis

Protein sample preparation including extraction, quantification and filter aided sample preparation (FASP) was according to the recommendations detailed in the iTRAQ kit (AB Sciex). Peptides were labeled with respective isobaric tags of 4-plex iTRAQ Reagent Multi-Plex kit (AB Sciex, PN: 4352135) according to the manufacturer supplied protocol (Applied Biosystems, MA, USA). Reverse phase LC-MS/MS analyzed using Thermo Scientific EASY-nLC 1000 System (Nano HPLC) and the raw data processed using Proteome Discoverer 1.3 Software. Proteins with a fold change ≥1.2 were considered significant as P value <0.01 and the false discovery rates (FDR) <0.01.

### Construction and analysis of protein–protein interaction (PPI) network

The PPI is considered as a basic skeleton for analysis of living organism self-organization and homeostasis [12]. Changed mRNA expressions and protein expressions based on the results of microarray and proteomics in high-protein diet rat model with strictly controlled urine concentrating ability were considered as the seed molecules, from which we obtained two direct and indirect PPIs using Bisogenet [13], a cytoscape [14] plugin. This method allows searching molecular interactions from well-known interaction databases including the Database of Interacting Proteins (DIP), the General Repository for Interaction Datasets (BIOGRID), the Human Protein Reference Database (HPRD), the Biomolecular Interaction Network Database (BIND), the Molecular Interactions Database (MINT) and the Database system and analysis tools for protein interaction data (IntAct)[15]. Then an intersection sub-network with nodes and edges merged by the two networks was obtained using the Advanced Network Merge plugin. In this network, each node represents a gene/protein and edge stands for the interaction between the two nodes.

### Identification of functional modules in the network

Clusters in a PPI network are often protein complexes and parts of pathways, while clusters in a protein similarity network represent protein families [16]. The Molecular Complex Detection (MCODE) [17] effectively finds closely connected regions of a molecular interaction network, many of which correspond to known molecular complexes, based solely on connectivity data. In this study, we used the MCODE to identify the functional modules with the degree cut-off = 2, K-core = 2 and Max.depth = 100. Six highly connected modules with smallest P value were detected.

### Functional enrichment analysis of the network

Intersection network and modules analysis: the KEGG (Kyoto Encyclopedia of Genes and Genomes) pathway enrichment and the gene ontology (GO) functional enrichment analysis were performed by using the online tool DAVID (Database for Annotation, Visualization, and Integrated Discovery) [18] based on the “KEGG_PATHWAY” and ‘‘GOTERM_BP_FAT’’ options. The terms only with P value <0.01 and FDR <0.05 were selected.

Analysis of the changed mRNAs and proteins: to further understand the biological relevance between the changed mRNAs or proteins and their regulators in the intersection sub-network, we performed functional enrichment analysis of these 106 changed mRNAs/proteins using Bingo [19], a plugin of the cytoscape.

### RT-qPCR

Total RNA of rat bladder urothelium was isolated by homogenization in TRIzol reagent (Invitrogen, Carlsbad, CA), cDNA was reverse transcribed from mRNA according to the instructions of RevertAid First Strand cDNA Synthesis Kit (Invitrogen). SyBR Green was used for fluorescence detection and real-time qPCR was carried out on Mxpro system. The specific mRNA amount was normalized by the β-actin amount from the same cDNA sample.

The primers used were as follows: EPHX2, F: 5′-CCTACTTGGCGCTTTCCAGAT-3′, R: 5′-GCAGCTTTCATCCATGAGTGG-3′; CDKN1C, F: 5′-AGAACAAGGCGTCGAACGAT-3′, R: 5′-GCAAGTTCTCTCTGGCCGTT-3′; CDK6, F: 5′-AGTGTTGGCTGCATCTTTGC-3′, R: 5′-CCTGTCTGGGAAGAGCAACA-3′; PCNA, F: 5′-AGAGCATGGATTCGTCTCACG-3′, R: 5′-TGGACATGCTGGTGAGGTTC-3′; BAX, F: 5′-TGGCGATGAACTGGACAACA-3′, R: 5′-CAGTTGAAGTTGCCGTCTGC-3′; MAGEB16, F: 5′-GCCAAGGTTCATGGGTCAGA-3′, R: 5′-CTCGGGCCAGCTTGTTCTAA-3′; SERPINE1, F:5′-CGTCTTCCTCCACAGCCATT-3′, R: 5′-GCTGGCCCATGAAGAGGATT-3′; HSPA2, F: 5′-CCTAACGTTGCTTTGCCTGT-3′, R: 5′-CACCTTGCCATGTTGGAAGAC-3′; FOS, F: 5′-AGACGAGAAGTCTGCGTTGC-3′, R: 5′-TCCAGGGAGGTCACAGACAT-3′; CRP, F: 5′-GCCTTCGTATTTCCCGGAGT-3′, R: 5′-ACATCAGCGTGGGCATAGAG-3′; SERPING1 F: 5′-CGTGGCCCGAAACTTACTCA-3′, R: 5′-TGGCAGTGCTTACTCAAGCC-3′; SRGN, F: 5′-GTTCAAGGTTATCCTGCTCGGA-3′, R: 5′-GGAAGAAATCATTCGGGAATCCTC-3′; MCPT9, F: 5′-ATTCCGGTTGGCCTGTCCTA-3′, R: 5′-TCTCGTGAGGTTTGGCCTTT-3′; CCNB1, F: 5′-TCCCACACGGAGGAATCTCT-3′, R: 5′-TCTGCAGACGAGGTAGTCCA-3′; MCPT2, F: 5′-TCCCACAACTTTAAGAGCAGCA-3′, R: 5′-TGGAGACTCGGGTGAAGATTG-3′.

### Histology and immunohistochemistry

4 % paraformaldehyde-fixed and paraffin-embedded rat bladders were sectioned at a thickness of 6-μm and stained with hematoxylin and eosin. Immunohistochemistry was performed using standard methods. Briefly, the slides were dried at 60 °C for 2 h and deparaffinized in xylene. Endogenous peroxidase activity was eliminated by 3 % H_2_O_2_ in ddH_2_O and antigen was retrieved by citric acid buffer (pH 6.0) for 10 min at 95 °C. The sections were blocked with 5 % (w/v) goat serum albumin for 30 min at room temperature. Primary antibodies (BAX, SANTA, 1:50 dilution; PCNA, CST, 1:100 dilution; F4/80, CST, 1:100 dilution; C-FOS, CyclinB1, P27, PAI-1, Ruiying Biological, 1:100 dilution) were applied and incubated overnight at 4 °C followed by secondary antibody (Scicrest Biotech, 1:200) for 30 min at 37 °C. Diaminobenzidene (DAB, ZLI-9018, Zhong Shan Jin Qiao) was employed for 5 min as the chromogen. The numbers of positive cells or relative intensity in 5 sections per mouse from 6 mice were quantitated and averaged by Image Pro Plus 6.0 software.

### Statistical analyses

All results were represented as mean ± SEM. Data involving in two groups was analyzed using two-tailed student *t*-test. When more than two experimental groups were compared, the data was analyzed using the Tukey-Kramer test with Prism 5.0 software to compare data between individual groups. P value <0.05 was considered to be statistically significant.

## Results

### Diet protein amount regulated urinary urea concentration and osmolality

**Fig. 1:**
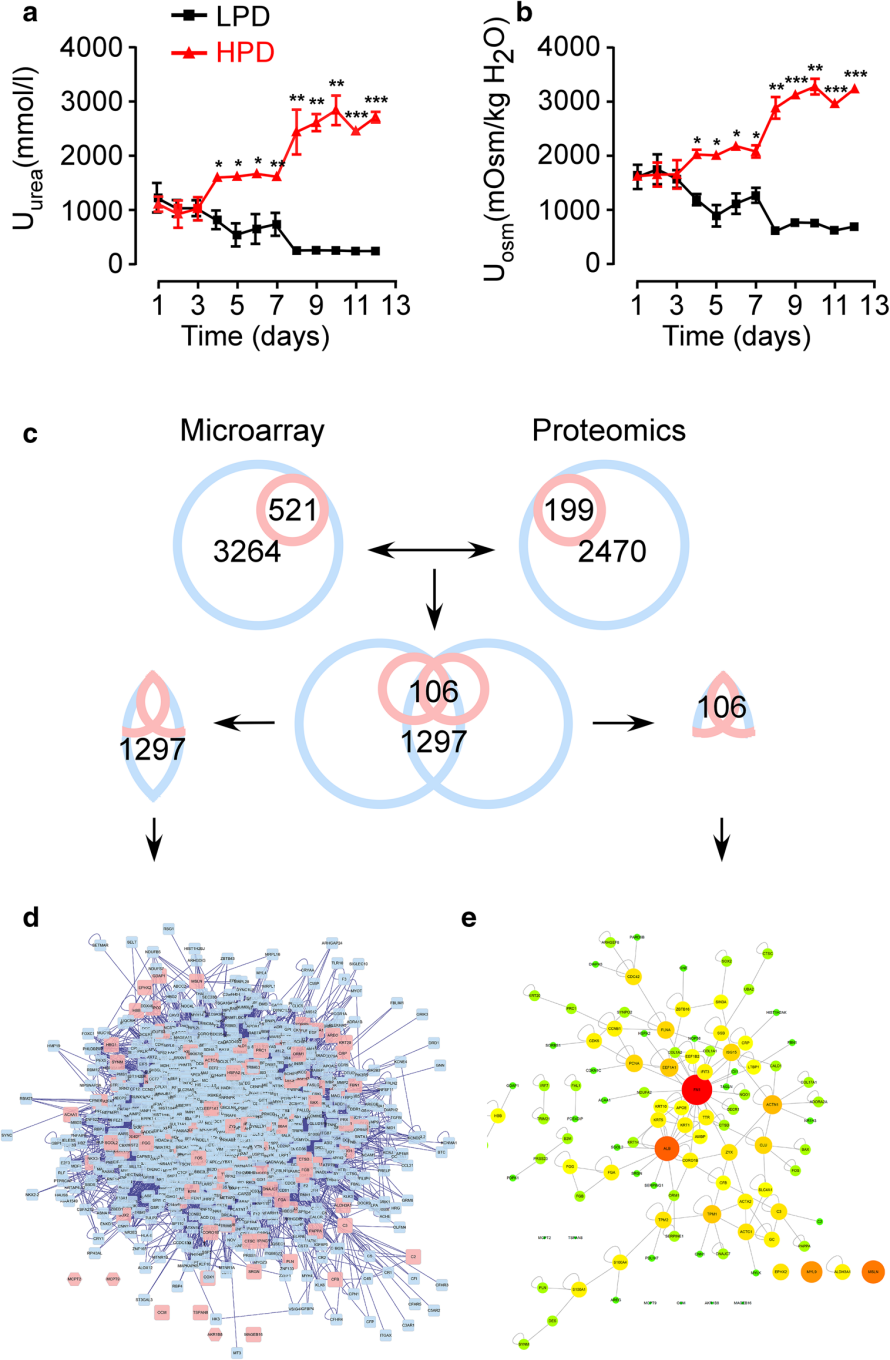
The levels of urea concentration, osmolality in urine and flow chart of network building. From day 1 to 3: normal diet with free access to water. Day 4–7: 40 or 10 % protein in diet in the high-protein diet group or low-protein diet, respectively. Day 8–12: 40 % protein in diet with 3.5 μg/kg/12 h of dDAVP injection in the HPD group and 10 % protein in diet with 10 ml/12 h water injection in the LPD group; **a** Urinary urea concentration. **b** Urinary osmolality. *n* = 6; Mean ± SEM.; *P < 0.05, **P < 0.01 and ***P < 0.001; NS stands for no significant. **c** Process of the combination of the two networks. **d** The intersection sub-network generated by the results of microarray and proteomics using Advanced Network Merge plugin*. Pink nodes* represent the seed molecules. *Blue nodes* stand for the interacting proteins generated by Bisogenet, based on the protein well-known interaction databases; **e** 106 nodes contained in the microarray and proteomics were derived from the intersection sub-network and considered as significant mRNAs/proteins

In rats fed with high-protein diet and treated with dDAVP, urea was highly concentrated in urine up to more than 2600 mmol/l, meanwhile, the urine osmolality was increased to almost 3000 mOsm/kg H_2_O (Fig. 1a, b). In rats fed with low-protein diet and treated with water, urinary urea concentration was decreased to approximately 150 mmol/l and urine osmolality decreased to around 700 mOsm/kg H_2_O. The urinary urea and osmolality in the high-protein diet group were almost 17- and 4-folds higher than those in the low-protein diet group, respectively.

### Diet protein amount regulated mRNA and protein expression in urothelium

Microarray and proteomics analysis of rat bladder urothelium showed levels of 521 mRNAs and 199 proteins changed in rats fed with different protein diet. These altered mRNAs and proteins were considered as the seed molecules, from which we obtained two PPI networks using Bisogenet, a Cytoscape plugin. There were 3264 nodes with 55,258 edges in the first network and 2470 nodes with 41,273 edges in the second network constructed by the results of microarray and proteomics, respectively (Fig. 1c). Then using the Advanced Network Merge plugin, an intersection sub-network with 1297 nodes and 2597 edges was obtained (Fig. 1d). Among these 1297 nodes, 106 nodes including 59 mRNAs (28 up-regulated and 31 down-regulated) significantly changed in the microarray and 67 proteins (47 up-regulated and 20 down-regulated) significantly changed in the proteomics, respectively, (20 nodes including FLNA, TTR, S100A4, TPM2, DES, C3, FGB, TAGLN, HBB, MSLN, CNN1, MYL9, EPHX2, ALDH3A1, MCPT9, MCPT2, MAGEB16, TSPAN8, AKR1B8, OCM were significantly changed in both), were considered as the significant mRNAs/proteins (Table 1). We mapped the nodes with high degree connectivity, analyzed by the network analyzer, to the large size with red color while the ones with low degree connectivity to the small size with green color inversely in Fig. 1e.

**Table 1:** 106 significant genes/proteins in the intersection sub-network

Gene symbol	Microarray (fold change)	Proteomics (fold change)	Degree	Module
MCPT9	*3.310011*	*1.510910*	0	
MCPT2	*2.780586*	*1.801877*	0	
HBB	*1.857657*	*1.367048*	14	
MAGEB16	*1.841369*	*1.219194*	0	
TTR	*1.738377*	*1.366377*	50	
ALDH3A1	*1.523775*	*1.347139*	4	
EPHX2	*1.513983*	*1.449821*	5	
FGB	*1.512520*	*1.217485*	19	
FLNA	*0.659427*	*1.428516*	146	
TSPAN8	*0.655180*	*0.817315*	0	
TPM2	*0.647158*	*1.677788*	38	
TAGLN	*0.647115*	*1.387740*	19	
AKR1B8	*0.636913*	*0.631030*	0	
MYL9	*0.632688*	*1.244950*	11	
DES	*0.596159*	*1.667755*	37	
OCM	*0.546350*	*0.772013*	0	
MSLN	*0.544640*	*0.617956*	13	
CNN1	*0.537661*	*1.430010*	13	
S100A4	*0.527200*	*0.800636*	40	
C3	*0.349307*	*1.553393*	32	
SERPINE1	*2.245071*	1.130098	15	
ISG15	*2.228321*	1.135855	81	B
SRGN	*1.915938*	1.123091	6	
IFIT3	*1.807260*	–	35	C
HSPA2	*1.740325*	1.055071	27	B
SOX2	*1.692999*	1.085868	26	B
ZBTB16	*1.684050*	1.096661	60	F
CDKN1C	*1.682264*	1.124969	10	
TPM1	*1.647525*	1.055467	45	
NR1H3	*1.632770*	–	20	
IRF7	*1.598710*	–	20	
COL17A1	*1.587436*	1.115462	9	
ADORA2A	*1.558627*	0.995801	9	
EEF1B2	*1.551863*	0.984599	40	C
HIST1H2AK	*1.536522*	1.036648	23	C
FOS	*1.524554*	1.119582	72	C
BAX	*1.520690*	1.115530	33	
HBG1	*1.515075*	–	10	
NDUFA2	*1.501249*	1.047733	20	
GDAP1	*1.501249*	1.025848	5	
FN1	*0.664729*	0.944520	386	A
PARD6B	*0.651307*	0.881654	14	
CCNB1	*0.640989*	0.920932	49	
DIAPH3	*0.634613*	0.983113	11	
SGOL2	*0.634613*	–	4	
PRC1	*0.629975*	0.980930	9	
ARHGEF6	*0.629887*	1.019215	23	
UBA2	*0.627761*	–	36	
LTBP1	*0.605885*	0.978378	11	
PAPPA	*0.601551*	–	10	
CDK6	*0.600723*	0.977918	37	B
PLN	*0.584696*	0.954198	4	
AREG	*0.580722*	**–**	8	
PRSS23	*0.519846*	–	12	
SERPING1	*0.513943*	0.892042	9	
FHL1	*0.485693*	1.198227	24	
C2	*0.477574*	–	5	
ACTC1	*0.455956*	0.967525	41	
KRT14	*0.453798*	0.835215	36	
KRT1	0.928397	*2.156542*	45	C
CRP	1.281350	*1.930480*	16	
CALD1	0.698924	*1.708044*	17	B
KRT20	1.000797	*1.659203*	7	
KRT10	1.000000	*1.632362*	41	
PDE4DIP	0.951103	*1.536384*	21	
DECR1	1.120752	*1.427631*	8	
SYNM	0.723882	*1.415602*	9	
SYNPO2	0.697342	*1.405946*	10	
PDLIM7	0.876647	*1.396267*	27	
MYLK	0.884821	*1.386343*	17	
ACTA2	–	*1.338323*	47	
CTSD	0.967471	*1.325037*	21	
ZYX	1.000000	*1.317850*	62	
NQO1	1.081580	*1.313200*	11	
GC	1.104551	*1.304363*	10	
ACAA1	–	*1.300248*	11	
SLC4A1	0.968066	*1.297543*	10	
FBN1	1.013898	*1.287208*	11	
ALB	1.397036	*1.266113*	69	
ACTN1	1.127086	*1.262533*	69	
CFB	–	*1.259225*	7	
FGA	1.000000	*1.249999*	19	
S100A1	1.328431	*1.244232*	16	
FGG	1.050224	*1.243389*	9	
SORBS1	–	*1.240217*	24	
CTSC	1.108849	*1.232253*	6	
AMBP	1.127504	*1.224902*	11	
COL1A1	1.138096	*1.224453*	32	
COL1A2	1.057467	*1.220590*	18	
APOE	1.375960	*1.214975*	32	C
KRT5	–	*1.204095*	40	
NOP58	0.860563	*0.830641*	47	
SSB	0.969600	*0.828536*	49	
B2M	0.941857	*0.827784*	13	
TRIM21	1.222173	*0.827560*	40	
CDC42	0.959164	*0.821915*	87	
PDPK1	0.928232	*0.817906*	13	
SIN3A	1.023062	*0.800982*	101	C
IDI1	0.842511	*0.790994*	3	
CORO1B	0.898386	*0.789598*	16	
GNE	0.914991	*0.770752*	6	
EEF1A1	1.011713	*0.769277*	204	D
DNAJC7	0.993363	*0.764110*	21	
PCNA	0.897037	*0.748792*	127	C
ORM1	0.864442	*0.637926*	7	
CLU	0.848667	*0.479796*	50	

Italic texts stand for significant changed

### High-protein diet regulated mRNAs/proteins expression involved in bladder disorders

**Fig. 2:**
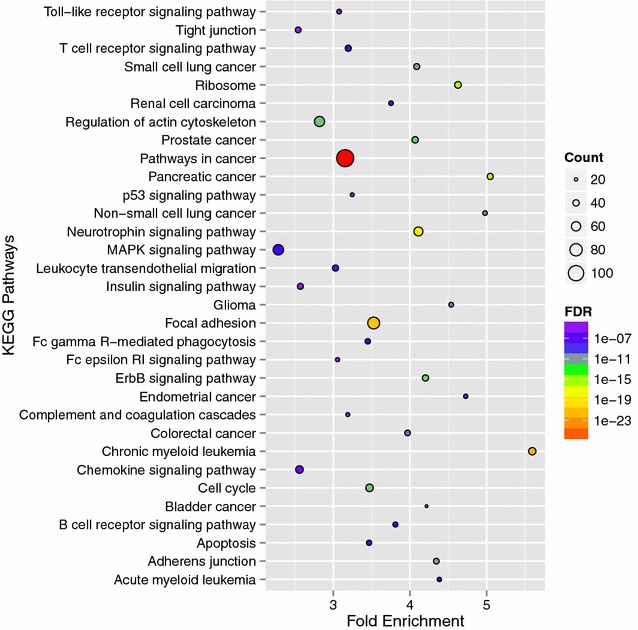
KEGG pathways of the intersection sub-network. *Bubble diagram* of the over-represented KEGG pathways of the intersection sub-network in bladder urothelium associating with high-protein diet was mapped using stats package in R environment

**Fig. 3:**
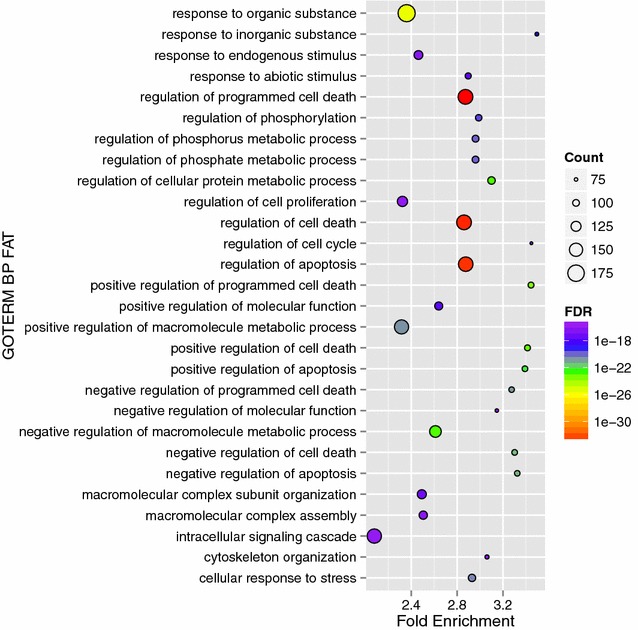
Gene ontology (GO) biological processes of the intersection sub-network. *Bubble diagram* of the over-represented gene ontology biological processes of the intersection sub-network in bladder urothelium associating with high-protein diet was mapped using stats package in R environment

To assess the predominant KEGG pathways and GO biological processes of this intersection network, we uploaded these 1297 mRNAs/proteins to the online biological classification tool DAVID and analyzed them based on the “KEGG_PATHWAY” and ‘‘GOTERM_BP_FAT’’ options. As shown in Figs. 2, 3, most of the KEGG pathways and biological processes focused on pathways in cancer, cell apoptosis, cell death and cell cycle: 112 mRNAs/proteins (9.67 %) were enriched in pathways in cancer with a fold enrichment score of 3.15; 49 mRNAs/proteins (4.23 %) were enriched in cell cycle regulation with a fold enrichment score of 3.47 (Fig. 2); 162 mRNAs/proteins (13.99 %) were enriched in cell death or apoptosis with a fold enrichment score of 2.86 (Fig. 3), etc.

**Fig. 4:**
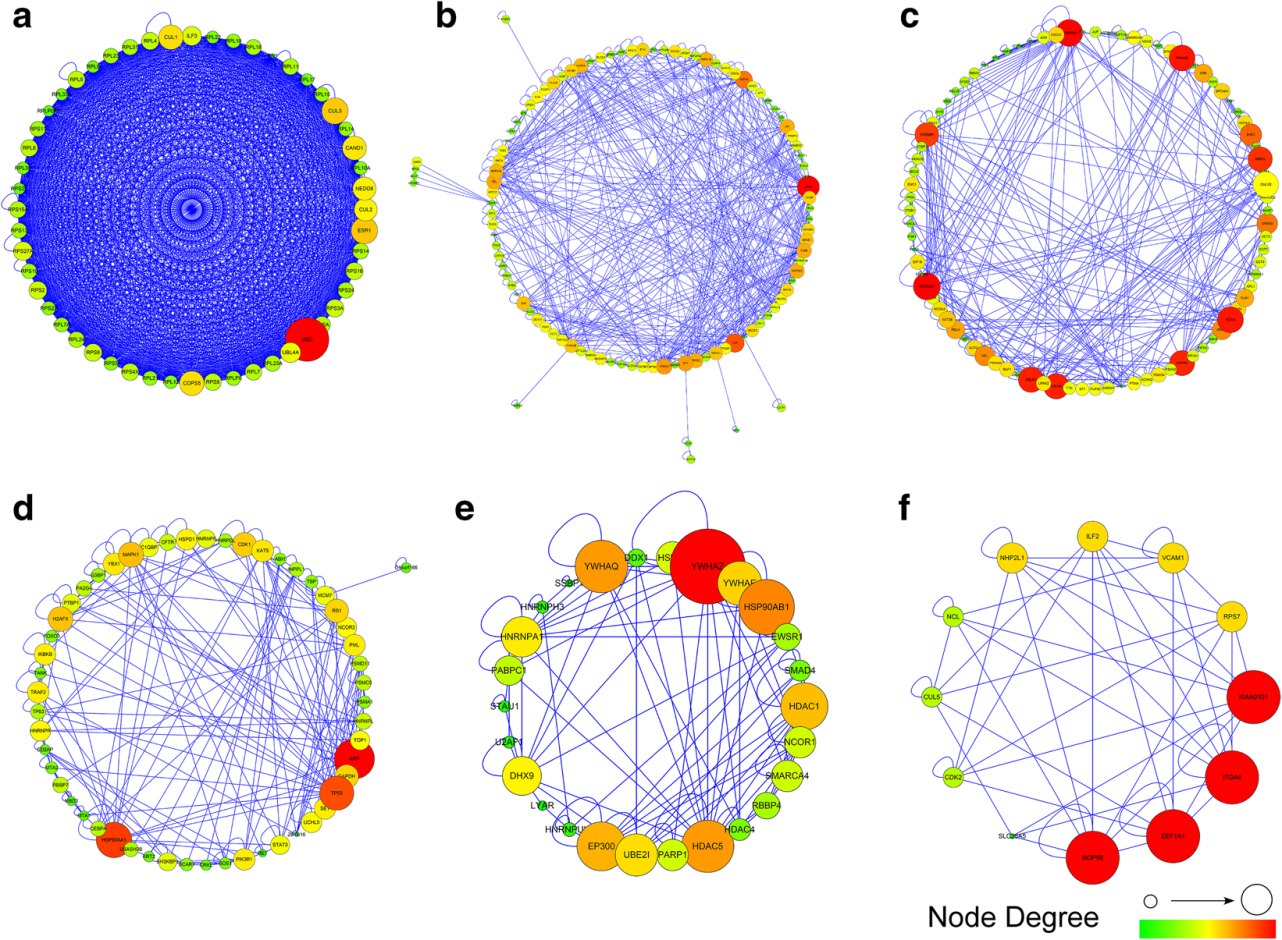
Functional modules identified by AllegroMCODE based on molecular complex detection (MCODE) algorithm. **a**–**f** Representative modules*. Circle dots* in the modules correspond to mRNAs/proteins. mRNAs/proteins in each module had similar biological functions: Module A was focused on ribosome biogenesis; module B was focused on pathways in cancer, T cell receptor signaling pathway, cell death and apoptosis; module C and D were focused on pathways in cancer; module E was focused on cell cycle; module F was focused on cell cycle and inflammation. *Large sizes* with *red color* stand for high degree connectivity, whereas *small sizes* with *green color* represent for low degree connectivity

To further identify the functional molecular complexes from this intersection sub-network, we employed a statistical approach of MCODE to cluster mRNAs/proteins. With degree >2, Degree Cut-off = 2, K-core = 2, Max.depth = 100, six modules were screened out (Fig. 4a–f). Module A including one (FN1, Fig. 4a) of these 106 significant mRNAs/proteins was focused on ribosome biogenesis; module B including five (ISG15, CDK6, HSPA2, SOX2, CALD1, Fig. 4b) mRNAs/proteins was focused on pathways in cancer, T cell receptor signaling pathway, cell death and apoptosis; module C including eight (PCNA, SIN3A, FOS, KRT1, EEF1B2, IFIT3, APOE, HIST1H2AK, Fig. 4c) mRNAs/proteins and module D including one (ZBTB16, Fig. 4d) mRNA/protein were focused on pathways in cancer; module E and F including zero mRNA/protein was focused on cell cycle (Fig. 4e, f) and inflammation (Fig. 4f), data were not shown. This clustering analysis further confirmed the activated biological processes from the KEGG pathway and GO analysis of the intersection sub-network.

**Fig. 5:**
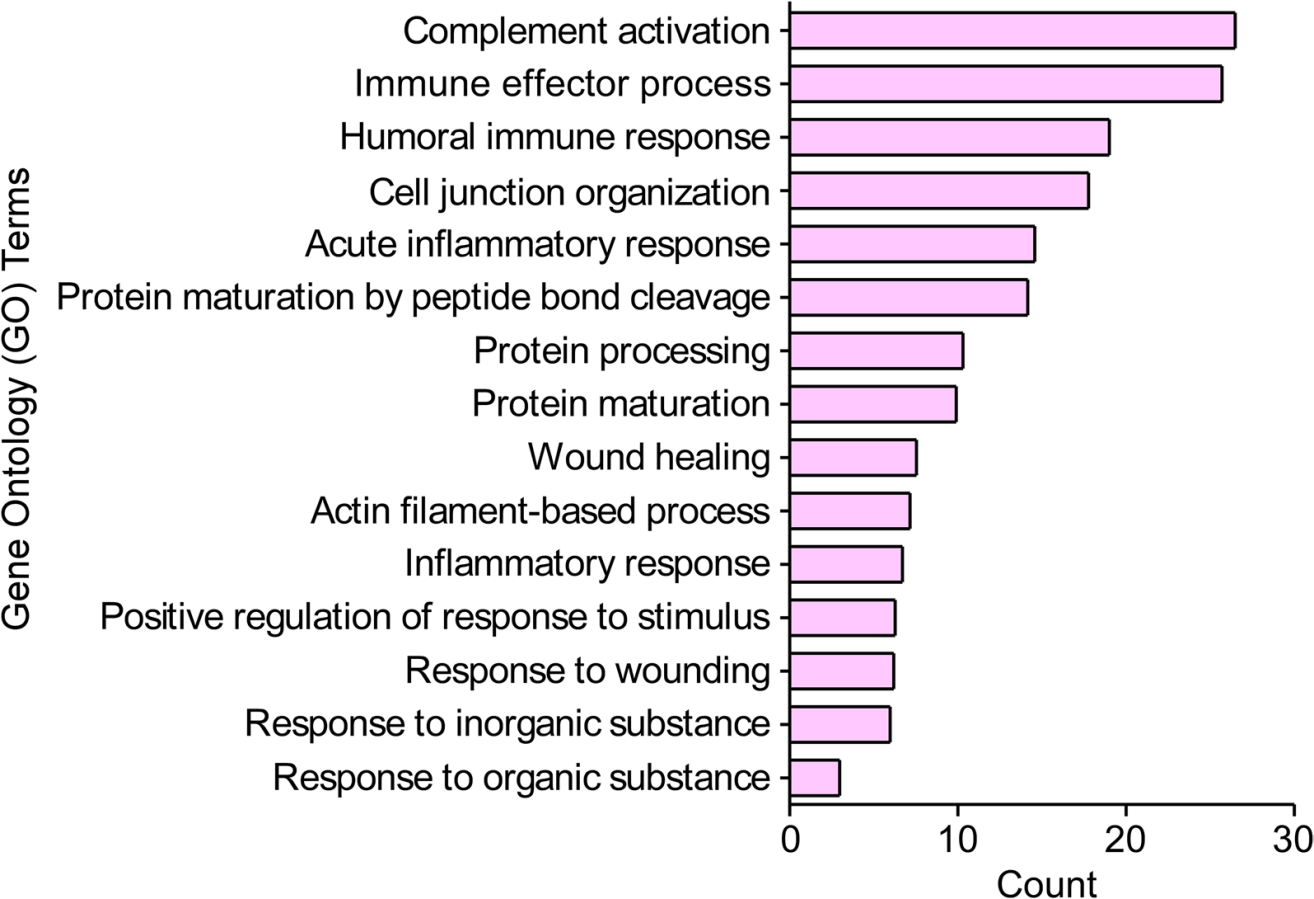
Gene ontology (GO) biological processes of the 106 significant mRNAs/proteins. The over-represented gene ontology biological processes of the 106 significant mRNAs/proteins in bladder urothelium associating with high-protein diet were analyzed using cytoscape plugin BINGO

Besides, to biologically characterize these 106 significant mRNAs/proteins in the intersection sub-network (Fig. 1e), we analyzed them using the Cytoscape plugin BINGO. The results revealed that these changed mRNAs/proteins had close relation with immune and inflammatory response (Fig. 5): 6 mRNAs/proteins (5.8 %) were enriched in complement activation, immune effector process and humoral immune response with a fold enrichment score of 26.49, 25.71 and 19.00 respectively, 9 mRNAs/proteins (8.7 %) were enriched in acute inflammatory response with a fold enrichment score of 14.57, etc.

Overall, based on the systemic signaling pathways and biological processes analysis of these six modules, 106 significant seed mRNAs/proteins and the intersection sub-network itself, immune and inflammatory response, cell cycle, cell death or apoptosis and especially cancer-related signaling pathways were highly associated with the effects of high urea concentration on the bladder urothelium. These data indicated that the changes in these signaling pathways and biological processes could be responsible for the pathological transition and lead to bladder disorders especially after persisting for a long time.

### Diet protein amount regulated biological pathways in bladder urothelium

**Fig. 6:**
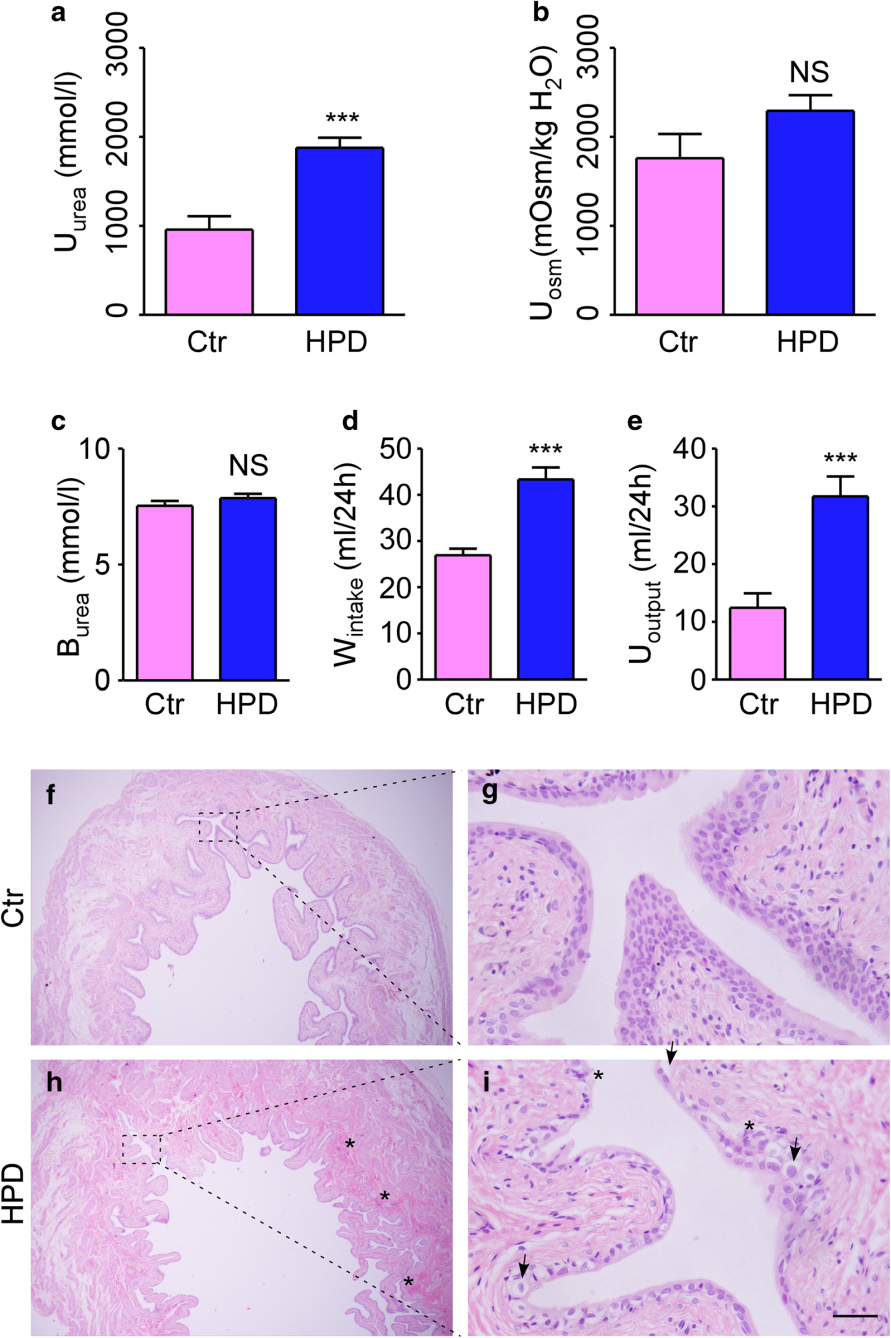
Biological effect of long term high-protein diet in rats. **a** Urinary urea concentration. **b** Urinary osmolality. **c** Blood urea. **d** Water consumption. **e** Urine output. **f** Representative magnification image of the bladder in control group. **g** High magnification image of *box* in f. **h** Representative magnification image of the bladder in HPD group. **i** High magnification image of *box* in h. *Asterisk* Interstitial congestion, inflammatory infiltrates, nucleus swelling and malformed. *Arrows* Endothelial cells thinned, desquamation, cytoplasm vacuolization

In order to further verify the effects of urinary urea concentration on biological processes in bladder urothelium, we carried out a long-term high-protein diet rat model in normal urine concentrating condition. After 40 days, urinary urea concentration was up to 1878 mmol/l in the HPD group that was twice higher than the normal-protein diet group (958 mmol/l). The urinary osmolality (2294 mOsm/kg H_2_O) was a little higher in the HPD group than that (1760 mOsm/kg H_2_O) in the normal-protein diet group in this model (Fig. 6a, b), which had no significant difference in statistics. Besides, there was no difference in the blood urea concentration between the two groups (Fig. 6c). The water consumption and urine output were much higher in HPD group than that in normal-diet group (Fig. 6d, e), indicating a high frequency of urination in the HPD group.

Hematoxylin and eosin (H and E) staining was adopted for morphologic analysis. As shown in Fig. 6f–i, after 40 days of high urea concentration stimulation on bladder urothelium caused by high-protein diet, the bladders showed interstitial congestion and inflammatory infiltrates under the urothelium in HPD group. Besides, endothelial cells desquamation, cytoplasm vacuolization, nucleus swelling and malformation also appeared in the HPD group.

**Fig. 7:**
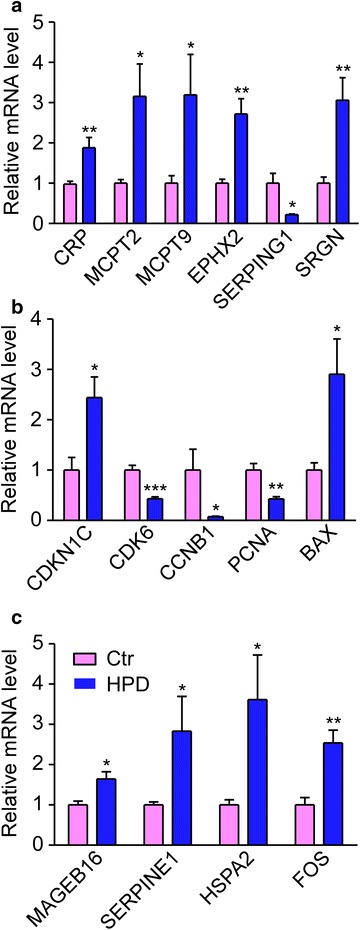
mRNA expressions of related genes in the over-represented biological processes. **a** Relative mRNA levels of genes involved in immune and inflammatory response. **b** Relative mRNA levels of genes involved in cell cycle regulation, cell death and apoptosis. **c** Relative mRNA levels of genes involved in pathways in cancer. All data were determined by fluorescence based qPCR. Mean ± SEM., *n* = 6

**Fig. 8:**
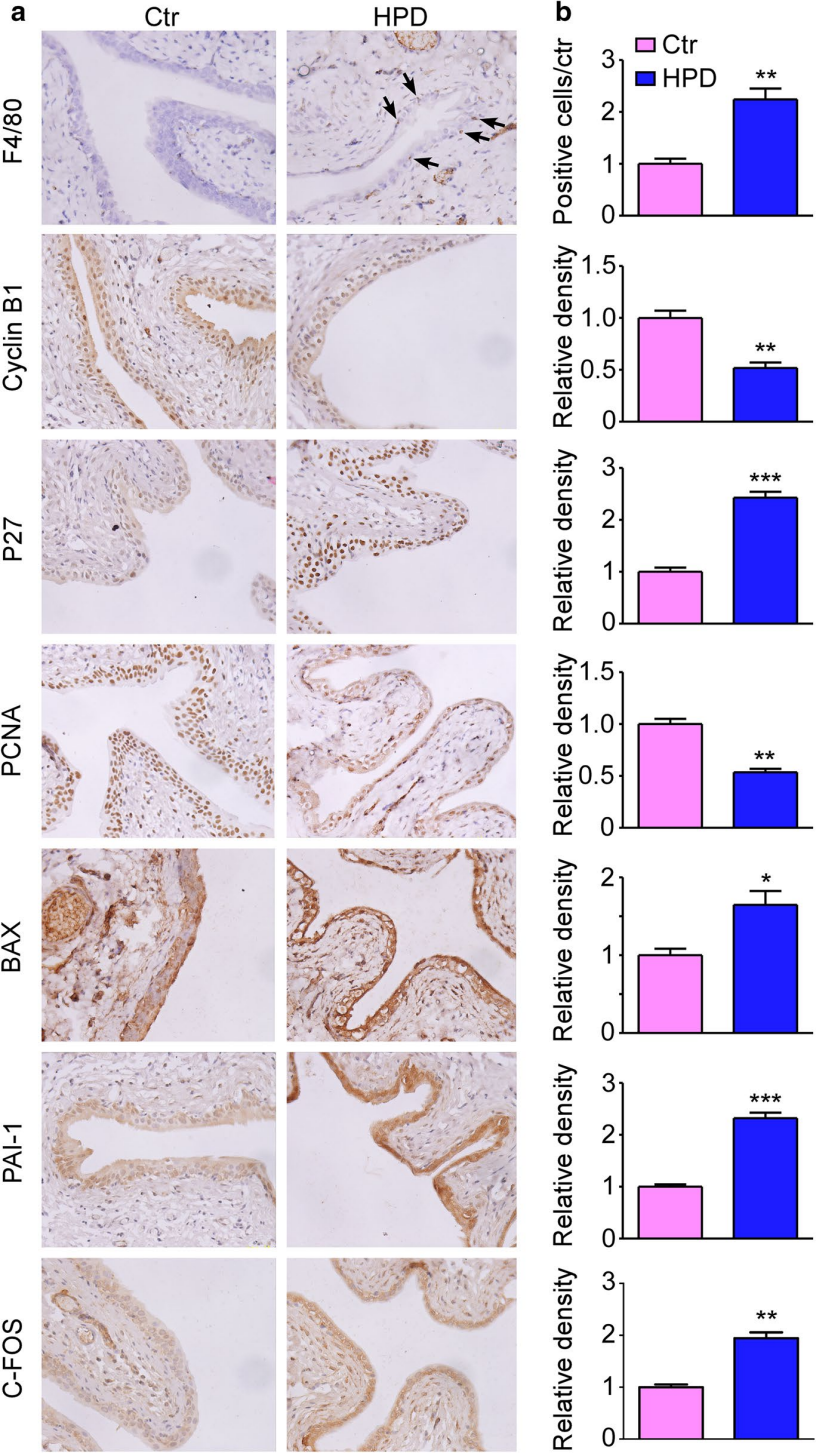
Immunohistochemistry of representative proteins in the over-represented biological processes. **a** Representative immunohistochemistry (*left*) of the bladder urothelium from the two groups. *Arrows* F4/80 positive cells. **b** *Bar graphs* showing the positive cells or density ratios of the representative proteins to control group. 5 sections per mouse from 6 mice were quantitated and averaged by Image Pro Plus 6.0 software. Mean ± SEM., *P < 0.05, **P < 0.01 and ***P < 0.001

Based on the analysis of the bioinformatics above, we concluded and tabled the significant mRNAs/proteins that obviously changed and were included in the immune and inflammatory response, cell cycle, apoptosis and especially cancer-related signaling pathways in Table 2. In the high-protein diet rat model, the qPCR results revealed that the mRNA expression of CRP, MCPT2, MCPT9, EPXH2, SERPING1 and SRGN, that were included in the immune and inflammatory response, was 1.88, 3.16, 3.20, 2.72, 0.22, 3.06 fold changed, respectively (Fig. 7a). Especially, the activation of this biological process was demonstrated by immunohistochemistry of the macrophage marker F4/80. As seen in Fig. 8a, macrophage infiltration was found under the bladder urothelium in the HPD group. The mRNA expression of CDKN1C, CDK6, CCNB1, PCNA, BAX, involved in the regulation of cell cycle and apoptosis, was 2.44, 0.43, 0.08, 0.43, 2.90 fold changed respectively (Fig. 7b). The cell cycle arrest and increased apoptosis were determined by the down-regulation of cyclin-dependent kinase (CCNB1/cyclin B1) and cyclin regulation protein (PCNA), and up-regulation of cyclin-dependent kinase inhibitor (CDKN1B/P27, a more representative protein than CDKN1C) and pro-apoptotic protein (BAX, Fig. 8a). Besides, the mRNA expression of MAGEB16, SERPINE1, HSPA2, FOS, involved in the cancer-related signaling pathways, was 1.64, 2.83, 3.62, 2.54 fold changed respectively (Fig. 7c). The protein expression of SERPINE1 (PAI-1) and FOS (C-FOS) was also detected by immunohistochemistry (Fig. 8a). The statistical results were shown on the right side of each picture in Fig. 8b.

**Table 2:** Highly identified biological processes and the corresponding genes/proteins

GO/pathway	Gene symbol	Microarray (fold change)	Proteomics (fold change)	Degree
Inflammatory response:	CRP	1.281350	*1.930480*	16
	MCPT2	*2.780586*	*1.801877*	0
	MCPT9	*3.310011*	*1.510910*	0
	EPHX2	*1.513983*	*1.449821*	5
	SERPING1	*0.513943*	0.892042	9
	SRGN	*1.915938*	1.123091	6
Cell cycle and apoptosis	CDKN1C	*1.682264*	1.124969	10
	CDK6	*0.600723*	0.977918	37
	CCNB1	*0.640989*	0.920932	49
	PCNA	0.897037	*0.748792*	127
	BAX	*1.520690*	1.115530	33
Pathways in cancer	MAGEB16	*1.841369*	*1.219194*	0
	SERPINE1	*2.245071*	1.130098	15
	HSPA2	*1.740325*	1.055071	27
	FOS	*1.524554*	1.119582	72

## Discussion

In the present study, we compared the two networks generated by the results of microarray and proteomics using Cytoscape. Gene ontology and pathway-based signaling regulator networks analysis revealed that immune and inflammatory response, cell cycle regulation, apoptosis, cell death and especially cancer-related signaling pathways were involved in the effects of high urea concentration on the bladder urothelium. These changed biological processes, which were further verified by qPCR, morphological and immunohistochemical staining, might lead to an increased risk of bladder disorders and even carcinogenesis:

In the high-protein diet group, inflammatory response related biological processes were highly identified. CRP (c-reactive protein), a reliable and widely used marker of systemic inflammation, was up-regulated in HPD group. Previous studies showed that blood CRP concentration was significantly higher in patients with urinary tract infection and interstitial cystitis [20, 21], which gained specific attentions in bladder dysfunction recently. Trichopoulos et al. found an association between the increased plasma CRP level and the risk of developing urothelial tumors. MCTP2 and MCPT9 (Mast cell protease 2 and 9) were up-regulated in HPD group. Mast cells are thought to play an important role in surveillance and maintenance of the body and regulate crucial biological process in innate and adaptive immunity [22]. Recent evidence indicated that MCPT2 and MCPT9 contributed to neutrophil recruitment and promoted the release of pro-inflammatory chemokines [23]. During most tumor initiation, these active inflammatory cells could induce the production or accumulation of reactive oxygen species (ROS) and reactive nitrogen intermediates (RNI), which resulted in DNA damage and genomic instability [24]. Another significant gene was EPHX2 (epoxide hydrolase 2) that was up-regulated in HPD group. Epoxyeicosatrienoic acids (EETs), the products of cytochrome P-450 epoxygenase metabolism of arachidonic acid, had been shown to prevent the activation of NF-κB that in turn led to activation of downstream inflammatory cytokines [25, 26]. However, soluble epoxide hydrolase (sEH) could metabolize it to their much less active dihydroxy derivatives dihydroxyeicosatrienoic acids (DHETs) and performed its pro-inflammatory functions. Besides, SERPING1, a C1-inhibitor, was down-regulated in the HPD group. Broadly speaking, C1 inhibitor plays important roles in a large amount of inflammatory diseases because of its suppression of inflammation through several activities including suppressing complement system proteases (e.g.: C1r, C1s, MASP2), leukocyte infiltration and expression of pro-inflammatory cytokines. Moreover, SRGN, encoding serglycin, was also up-regulated in the HPD group. Previous studies demonstrated that serglycin played important functional roles in immune response, inflammation and even tumorigenesis. Serving as an ideal molecular partner for multiple molecular effectors, it could regulate the biosynthesis, secretion and targeted delivery of many inflammatory mediators including growth factors, cytokines, and chemokines to specific target sites. These functions turned to enhance inflammatory process and support tumor growth and metastasis [27].

It is now well elaborated that inflammatory microenvironment is highly associated with tumorigenesis. In this model, active inflammatory response were identified along with up-regulated of these pro-inflammatory mRNAs/proteins and down-regulated of anti-inflammatory ones, which revealed that inflammatory injury occurred in the rat bladder urothelium under the high urea concentration stress that would then increase the oncogenic mutation rates and raise the risk of bladder carcinogenesis.

The cell cycle is controlled by many regulators via a variety of mechanisms to ensure the alternation of each phase in order. It is now well established that cyclin-dependent kinase (CDKs), cyclin-dependent kinase inhibitor (CKIs) and cyclin are the three main categories of these regulators. Cyclically accumulation and decomposition of cyclin play a positive regulation function in cell cycle progression, whereas CKIs play a negative regulation function through inhibiting the CDKs in the appropriate point of cell cycle. The balance of these regulators working synergistically with multiple factors promotes the smooth process of cell evolution [28–30].

Under the stress of high urinary urea concentration, the expression of CDKN1C (cyclin-dependent kinase inhibitor 1C) was up-regulated in the bladder urothelium. On the contrary, the expression of CDK6, CCNB1 (a member of the highly conserved cyclin family) and PCNA (proliferating cell nuclear antigen,) was down-regulated. PCNA is critical in DNA synthesis, repair and cell cycle regulation and it is a good indicator reflecting the state of cell proliferation [31]. Recently, numerous evidences revealed a closely relationship between PCNA and tumorigenesis, development, metastasis, prognosis, etc. Besides, apoptosis-related protein BAX was increased in the HPD group. As a member of the Bcl-2 family proteins, BAX can promote apoptosis by translocating to the mitochondria membrane from cytoplasm and forming oligomers and then leads to the release of cytochrome c that triggers apoptosis.

The changes of these significant regulators in cell cycle and apoptosis-related proteins indicated that cell cycle arrest and increased apoptosis were induced by the stress of high urea concentration, which is consistent with the previous report that acute urea loading induced cell cycle delay and apoptosis [32]. These abnormal biological processes may lead to early injuries in urothelial cells or bladder disorders and hence increased the risk of bladder tumorigenesis.

Moreover, lots of cancer-associated mRNAs/proteins changed, which revealed a deterioration of the development tendency in the bladder urothelium. The most significant ones were MAGEB16 (Melanoma-associated antigen B16), SERPINE1 (Plasminogen activator inhibitor 1 RNA-binding protein, PAI-1), HSPA2 (Heat shock 70 kDa protein 2) and FOS (Proto-oncogene C-FOS), which were all up-regulated in the HPD group.

Plasminogen activator inhibitor 1 RNA-binding protein, an important member of the plasminogen activator system, was found to be elevated in various cancers and its high levels in tumors usually coincided with poor prognosis. Although the contribution of PAI-1 to tumor angiogenesis had long been controversial, it was recently accepted that it could promote cancer cell proliferation and angiogenesis by inhibiting apoptosis in cell culture systems [33]. Villadsen et al. found that miR-143/-145 cluster, which directly targeted the PAI-1 3’UTR and reduced PAI-1 mRNA and protein levels, was down-regulated in all stages of bladder cancer and inversely correlated with PAI-1 expression. So they suggested miR-145 and PAI-1 as clinically relevant biomarkers of the bladder cancer [34]. HSPA2, whose abnormal expression is usually found in a subset of cancers, has also been identified as a potential tumor-promoting protein. ManojGarg et al. found that HSP70-2 was expressed in both high-grade invasive and moderate to well-differentiated urothelial carcinoma cell lines. Down-regulation of HSP70-2 could decrease cell growth, colony formation, invasion and migration of urothelial carcinoma cells, which makes it as a potential therapeutic target [35]. C-FOS, which has oncogenic activity and plays a significant role in proliferation, differentiation, invasion, metastasis, and survival in tumor cells, is frequently over-expressed in many types of cancer [36]. Some studies also reported that C-FOS played an important role in arsenic-mediated carcinogenesis of the urothelium [37, 38]. In addition, Li et al. found that microRNA-490-5p inhibited the proliferation of bladder cancer by targeting the C-FOS 3’UTR and decreasing C-FOS expression at both mRNA and protein levels [39]. Overall, these mRNAs/proteins contained in the pathways in cancer were up-regulated and predicted a deterioration of the development tendency in the bladder urothelium.

Our previous work found that UT-B deletion in mice caused a marked urea accumulation in bladder urothelial cells, resulting in DNA damage and apoptosis. In accordance with other research groups, they had reported that high urea concentration could affect protein stability by destroying hydrophobic bonds of protein structure or causing protein carbamylation and then changed multiple biological activities [40, 41]. As the epidemiological studies showed that the incidence of bladder cancer was the highest in the developed countries of Western Europe and higher in men than women [42], we considered this high incidence might be linked to high urinary urea concentration because of their high-level consumption of lean meat, milk, eggs or other high-protein food [43].

In physiological context, urea can be highly concentrated in urine up to more than 1000 mmol/l, however, it is usually less than 10 mmol/l in blood. Urothelial cells serve as a barrier between urine and blood. Under normal conditions, urea concentration is about 20.0 mmol/l in the urothelium [11]. In our high-protein diet models, it could be much higher than the normal value (That can be speculated by the increased urea concentration in urinary), although we did not detect it because of the limited amount of sample and technical difficulty. Although this value is not seriously high, it could still create an imbalance between the arginine-ornithine-polyamine pathway and the arginine-citrulline-NO pathway as discussed in our previous study and increase protein carbamoylation and carbonylation that is attributed to oxidative stress.

However, it should be noted that the limitations do exist in our present works: Firstly, the rats fed with different protein diets were just experimental models to study the people who eat high-protein food over a long period of time. The experimental period was too short to simulate people in some clinical characterizations. Nevertheless, it revealed, to a certain extent, a relationship between high urea concentration and early injuries of urothelical cells or bladder disorders. Secondly, although urea was the major solute in urine under this condition and it could account for the bladder disorders directly, we could not exclude the possibilities of other indirect factors including the high osmolality itself. Thirdly, the main results in this study were based on the bioinformatics techniques and verified only by qPCR, H.E. and IHC. More other epidemiological and experimental studies should be practiced in the future.

## Conclusion

Our present work firstly elaborated the relation between the high-protein diet and the risk of bladder disorders. It showed that abnormally activated inflammatory response, cell cycle arrest, apoptosis and pathways in cancer occurred in bladder urothelium under the stress of high urinary urea concentration, which provides us a new hint to associate the inducement of bladder disorders and even carcinogenesis with high urinary urea concentration caused by high-protein diets.

## Abbreviations

UBC: urothelial bladder cancer
HPD: high-protein diet group
LPD: low-protein diet group
NPD: normal-protein diet group
UT-B: urea transporter B
*slc14a1*: solute carrier family 14, member 1
MCODE: molecular complex detection
KEGG: Kyoto encyclopedia of genes and genomes
GO: gene ontology
DAVID: database for annotation, visualization, and integrated discovery
